# Tumour Colonisation of *Parvimonas micra* Is Associated with Decreased Survival in Colorectal Cancer Patients

**DOI:** 10.3390/cancers14235937

**Published:** 2022-11-30

**Authors:** Thyra Löwenmark, Anna Löfgren-Burström, Carl Zingmark, Ingrid Ljuslinder, Michael Dahlberg, Sofia Edin, Richard Palmqvist

**Affiliations:** 1 Department of Medical Biosciences, Pathology, Umeå University, SE-90185 Umeå, Sweden; 2 Department of Radiation Sciences, Oncology, Umeå University, SE-90185 Umeå, Sweden; 3 Department of Surgical and Perioperative Sciences, Surgery, Umeå University, SE-90185 Umeå, Sweden

**Keywords:** mucosal microbiota, *Parvimonas micra*, *Fusobacterium nucleatum*, survival, colorectal cancer

## Abstract

**Simple Summary:**

The gut microbiota has been suggested to affect tumour development and progression in colorectal cancer. In this study, we investigated the associations of two colorectal cancer-related bacteria, *Parvimonas micra* and *Fusobacterium nucleatum*, with survival in colorectal cancer patients. Our results suggest that patients with high tumoural levels of *Parvimonas micra* have decreased survival. In addition, we found associations for *Parvimonas micra* and *Fusobacterium nucleatum* with different clinicopathological and tumour molecular traits. A better understanding of the role of the gut microbiota in colorectal cancer may contribute to improved cancer care.

**Abstract:**

Increasing evidence suggests that the gut microbiota may impact colorectal cancer (CRC) development and progression. In this study, the tumour colonisation of two CRC-associated bacteria, *Parvimonas micra* and *Fusobacterium nucleatum*, was studied in relation to patient survival in a cohort of 257 CRC patients. Colonisation of *P. micra* and *F. nucleatum* was analysed in fresh frozen tumour tissue (n = 112) and in faeces (n = 250) by qPCR. When analysing tumour tissues, both *P. micra* and *F. nucleatum* were found to be associated with decreased five-year cancer-specific survival, an association that remained significant in multivariable analysis for *P. micra*. Furthermore, we found significant associations of high levels of *P. micra* and *F. nucleatum* with tumour molecular characteristics, i.e., tumours mutated in *BRAF^V600E^*, and tumours of the MSI subtype. The analysis of faecal samples showed weaker associations with prognosis and tumour molecular characteristics. In conclusion, our findings support a novel association of tumour colonisation of *P. micra* with decreased patient survival. A better understanding of the role of the gut microbiota in CRC might contribute to the advancement of prognostic tools and new targets for therapy.

## 1. Introduction

Colorectal cancer (CRC) is one of the most deadly cancers in both men and women worldwide with close to 1 million deaths per year, and the number is expected to rise due to an increasing incidence in younger adults and transitioning countries [1]. CRC is a disease with high heterogeneity where patient prognosis depends on a complex interplay between environmental, molecular and genetic factors. Rising evidence suggests gut dysbiosis plays a crucial role in CRC development and progression and metagenomic studies have revealed a structural segregation in microbial composition between CRC patients and healthy individuals [2]. A driver-passenger theory has been introduced, where pro-oncogenic ‘alpha-bugs’ possess the potential to outcompete other cancer-protective species and drive tumorigenesis through immune response remodulation and the production of toxins, leading to cell damage. Disease progression in turn changes the gut microenvironment, resulting in a shift in the microbial community [3]. As a result, the initial bacterial drivers are gradually replaced with gut commensals that can promote tumour progression through inflammatory processes [3]. The driver-passenger theory has been supported by numerous studies on the role of the gut microbiota in CRC, including mechanisms of genotoxin-induced tumourigenesis, and mechanisms controlling barrier function and inflammation [4]. Inflammation in fact plays dual roles in CRC carcinogensis, where on the one hand it may act protective in response to acute tissue damage and display anti-tumourigenic effects, and on the other hand, chronic inflammatory processes are known to induce tumour growth and progression [4,5].

Together, this makes the gut microbiota a potential and interesting target for future cancer therapy. Indeed, recent studies have suggested that the intestinal microecological composition is related to CRC prognosis [2] and specific gut microbial species have been associated with a deteriorated response to cancer therapy [6]. However, the relationship between the spatio-temporal presence of CRC-related microbes and the functional aspects behind clinical outcome is still largely unknown.

Interestingly, many of the bacterial species suggested to be associated with CRC are often found colonising the oral microflora, one of the more well-studied being *Fusobacterium nucleatum* [7,8,9,10,11,12]. *F. nucleatum* has been suggested to contribute to colorectal tumour progression through various mechanisms, including expression of the virulence factor FadA on the cell surface, which induces E-cadherin mediated activation of Wnt/β-catenin signalling [13]. Previous studies have also proposed an association between gut colonisation of *F. nucleatum* and decreased CRC survival [14,15,16,17,18,19,20,21]. Another oral pathogen suggested to be associated with CRC is *Parvimonas micra* [9,10,11,12,22,23,24]. In a previous study using specific qPCR, we found both *P. micra* and *F. nucleatum* to be more abundant in stool samples from CRC patients compared with healthy individuals [23]. The analysis of fresh frozen tumour tissues enabled us to link *P. micra* to tumour immunological traits [25]. A recent study on mice suggests *P. micra* has a tumour-promoting effect through colonocyte proliferation and the alteration of the Th17 immune response [26]. Still, in contrast to *F. nucleatum*, very little is known about the potential role of *P. micra* in CRC tumourgenesis and prognosis.

In this study, we investigated if the levels of *P. micra* and *F. nucleatum* in tumour tissue and faeces of CRC patients affect cancer-specific survival. A potential link between microbial colonisation in CRC and patient prognosis would facilitate the development of new prognostic and predictive tools, as well as future targeted cancer therapies.

## 2. Materials and Methods

### 2.1. Study Cohort

The study was performed on patient samples collected within the Uppsala-Umeå Comprehensive Cancer Consortium (U-CAN), which has been described previously [27]. The project longitudinally collects blood, tissue, faeces, radiological data and clinical data from patients diagnosed with CRC. The fresh frozen tissue, formalin-fixed paraffin-embedded (FFPE) tissue and faecal samples included in this study were collected at Umeå University Hospital between the years 2010–2014. During this period, a total of 684 patients were included in the study, of which 260 Stage I–IV CRC patients (38%) provided a stool sample before start of treatment. The procedure for the faecal sample collection has been described previously [23]. Since that time, a few patients (n = 3) decided to withdraw their participation in the study and their samples have not been included, leaving in total 257 CRC patients with collected faecal samples. Of the patients with collected faecal samples, fresh frozen tumour tissues were available from 115 patients, and FFPE tissues from an additional 72 patients biopsied or surgically resected at Umeå University Hospital. No FFPE material was accessible for patients included in the study but operated on at another hospital (n = 59).

### 2.2. Detection of Microbial Markers in Fresh Frozen Tumour Tissue and Faeces Using Quantitative Real-Time PCR (qPCR)

Detection of *P. micra* and *F. nucleatum* in fresh frozen tumour tissue and faeces was conducted using qPCR as described previously [23,25]. In brief, a 2–3 mm cube of fresh frozen tumour tissue was homogenised using the Precellys^®^ Soft Tissue Homogenizing CK14 Kit (Bertin Techologies, Rockville, MD, USA). The AllPrep DNA/RNA/miRNA Universal kit (Qiagen, Sollentuna, Sweden) was next used for DNA extraction. For faeces, the QIAamp PowerFecal DNA kit (Qiagen) was used to extract DNA from about 0.2 g stool. The Qubit dsDNA BR Assay Kit (Invitrogen, Carlsbad, CA, USA) was used to measure DNA concentration. The specific qPCR assays used to detect *P. micra*, *F. nucleatum*, and relevant reference genes were previously established [23,25]. The Quant-Studio™ 6 Flex Real-Time PCR System (Applied Biosystems, Foster City, CA, USA) was used for qPCR reactions. Data from stable duplicates was presented. Markers not amplified within 38 cycles were defined as negative. Samples with discrepancies in Cq values (standard deviation > 0.5) between duplicates were rerun, but after three discordant runs excluded. Further exclusions included depleted samples or poor sample quality. For fresh frozen tumour tissues, 3 samples were excluded after the control *PGT* assay, leaving 112 samples for further analyses. Additional exclusions included 2 samples for *P. micra* and 2 samples for *F. nucleatum*. For the faecal samples, 7 samples were excluded after the 16S rRNA gene analysis, leaving 250 samples for further analysis. For *P. micra* analysis, 4 additional samples were excluded and for *F. nucleatum*, 6 additional samples were excluded from analyses. The levels of *P. micra* and *F. nucleatum* were presented as a relative quantification using the 2^−ΔCq^ method. The human gene *PGT* was used as reference for fresh frozen tissue and the 16S rRNA gene was used as reference for faeces.

### 2.3. Molecular Analyses

#### 2.3.1. Microsatellite Instability (MSI) Assessment

Molecular analyses were performed on DNA extracted from fresh frozen tumour tissues as described above. For patients lacking fresh frozen tumour tissue specimens, DNA was instead extracted from FFPE tumour tissues (five sections of 10 µm) using the AllPrep DNA/RNA FFPE kit (Qiagen) (n = 72). For determination of MSI status, the MSI Analysis System Version 1.2 (Promega) was used, based on the analyses of mononucleotide repeat markers BAT-25, BAT-26, NR-21, NR-24, MONO-27, as was previously described [28]. The Peak Scanner™ Software v1.0 (Applied Biosystems) was used for analyses of data. When two or more markers were altered, the tumours were classified as MSI. Remaining samples were classified as MSS.

#### 2.3.2. Analyses of BRAF and KRAS Mutation

For analyses of *BRAF^V600E^* mutation, digital droplet PCR (ddPCR, Bio-Rad Laboratories) was used, as described previously [28]. In brief, PCR was performed with 900 nM of the primers and 250 nM of each probe in a total volume of 20 µL divided into 20,000 nanolitre droplets using a T100 Thermal Cycler (Bio-Rad Laboratories, Hercules, CA, USA). The following programme was used for amplification: 95 °C for 10 min; 40× cycles of 95 °C for 15 s; 56 °C for 1 min (ramp rate 2 °C/s) and 98 °C for 10 min. For *KRAS* mutation analysis, codon 12 and 13 were sequenced using Big Dye v.3.1 (Applied Biosystems). Primers and probes for *BRAF* and *KRAS* mutation analyses were described previously [28].

### 2.4. Statistical Methods

IBM SPSS Statistics 28 (SPSS Inc., Chicago, IL, USA) was used for statistical analyses. *p*-values < 0.05 were considered statistically significant. Correlations between continuous variables were analysed using the Spearman’s rank correlation tests. For comparisons of categorical variables, Fisher’s exact test was used.

Included patients were followed from the date of surgery until the date of death or end of follow-up (October 2021). Cancer-specific survival was defined as death with disseminated or recurrent disease. Patients not undergoing resection of the primary tumour, and patients who died from post-operative complications within 90 days, were excluded from survival analyses. ROC curves were created using the relative levels of *P. micra* or *F. nucleatum* as test variable and cancer-specific survival as state variable. Youden’s index was then used to calculate the optimal cut-off to identify samples with high or low levels of the markers.

Kaplan–Meier plots were used to estimate cancer-specific survival over time and the log-rank test was used to calculate statistical differences in outcome between groups. Survival analyses were truncated at 5 years to fulfil the assumption of proportional hazards. Uni- and multivariable survival analyses were performed using the Cox Proportional Hazards model. Variables included in the multivariable analyses were selected using (1) Backward Stepwise Conditional model and (2) only including variables with a significant effect on survival in univariable analysis, including all available clinicopathological and molecular variables. Both methods resulted in the same variables being included in the multivariable analyses (stage, grade and levels of *P. micra* or *F. nucleatum*). Since the microbial variables included in the study were correlated, separate multivariable analyses were conducted for these.

## 3. Results

### 3.1. Distribution of P. micra and F. nucleatum in Tumour Tissue and Faeces of CRC Patients

The levels of *P. micra* and *F. nucleatum* were evaluated by qPCR in fresh frozen tumour tissues (n = 112) and in faecal samples (n = 250) from a CRC patient cohort. For fresh frozen tumour tissues, relative quantification of *P. micra* and *F. nucleatum* was calculated using the human gene *PGT* as reference. *P. micra* was detected in tumour tissue from 30 out of 110 (27.3%) patients and *F. nucleatum* in 69 out of 110 patients (62.7%). For faecal samples, the relative levels of *P. micra* and *F. nucleatum* were calculated using the 16S rRNA gene as reference. *P. micra* and *F. nucleatum* were detected in faecal samples from 143 out of 246 (58.1%) and 185 out of 244 patients (75.8%), respectively.

**Figure 1:**
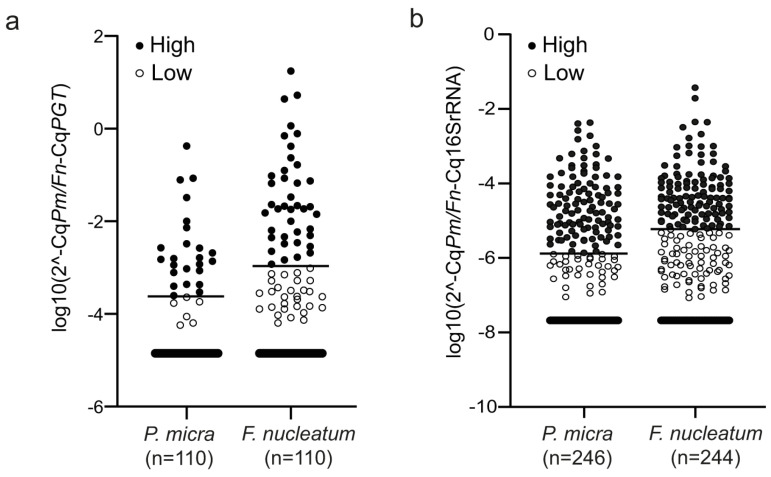
The levels of *P. micra* and *F. nucleatum* in CRC patients. Scatter plots illustrating the relative levels of *P. micra* (*Pm*) and *F. nucleatum* (*Fn*) in (**a**) fresh frozen tumour tissue and (**b**) faeces. Horizontal lines indicate the cut-off between high and low levels of the bacteria as calculated by the 2^−ΔCq^ method using either *PGT* (for fresh frozen tumour tissue) or 16s RNA (for faeces) as reference.

ROC curve analyses based on cancer-specific survival were used to calculate an optimal cut-off value for high or low levels of *P. micra* and *F. nucleatum*. The distribution of samples classified as high and low can be found in Figure 1 and Table 1. Utilising this cut-off, 24 out of 110 (21.8%) patients were identified with high levels of *P. micra* and 40 out of 110 patients (36.4%) with high levels of *F. nucleatum* in tumour tissue. In faeces, 108 out of 246 patients (43.9%) had high levels of *P. micra* and 114 out of 244 patients (46.7%) had high levels of *F. nucleatum*. Of the 24 patients with high levels of *P. micra* in tumour tissue, 20 patients also had high levels of *P. micra* in faeces (Table 1). For *F. nucleatum* analysis in patients with both tumour tissue and faeces available, 24 out of 39 patients with high levels in tumour tissue also had high levels in faeces (Table 1). Furthermore, there was a significant correlation between the relative levels of *P. micra* or *F. nucleatum* in tumour tissue and in faeces (r_s_ = 0.533, *p* < 0.001 for *P. micra*; r_s_ = 0.468, *p* < 0.001 for *F. nucleatum*).

**Figure 2:**
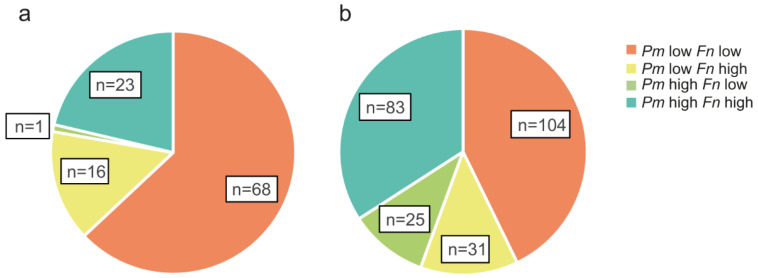
The relative distribution of *P. micra* and *F. nucleatum* in CRC patients. Pie charts are used to illustrate groups of patients with high or low levels of *P. micra* (*Pm*) and/or *F. nucleatum* (*Fn*) in (**a**) fresh frozen tumour tissue (n = 108) or (**b**) faeces (n = 243) of patients analysed for both microbial markers.

Interestingly, 23 of the 24 tumour tissues with high levels of *P. micra* also had high levels of *F. nucleatum* (Figure 2). In faeces, 83 of the 108 samples with high levels of *P. micra* also had high levels of *F. nucleatum* (Figure 2). Moreover, there was a significant positive correlation between the levels of *P. micra* and *F. nucleatum* in tumour tissue (r_s_ = 0.561, *p* < 0.001) and in faeces (r_s_ = 0.640, *p* < 0.001).

### 3.2. Association of P. micra and F. nucleatum in Tumour Tissue and Faeces with Clinicopathological and Molecular Parameters

The relations of *P. micra* and *F. nucleatum* to clinicopathological and molecular characteristics of the study patients were analysed using the cut-off for high/low levels (Table 2 and Table 3, respectively, for fresh frozen tissue). High levels of *P. micra* in tumour tissue were significantly associated with age and female gender (Table 2), as well as *BRAF*-mutated tumours and tumours of the MSI subtype (Table 3). As with *P. micra*, high levels of *F. nucleatum* were significantly associated with age (Table 2), as well as *BRAF*-mutated tumours and tumours of the MSI subtype (Table 3). In addition, *F. nucleatum* was significantly associated with right-sided tumours (Table 2). In faeces, when all included clinical and molecular parameters were analysed, the only significant associations found were those of *P. micra* and *F. nucleatum* with tumours of the MSI subtype (Supplementary Tables S1 and S2).

### 3.3. High Levels of P. micra and F. nucleatum in Tumour Tissue Are Associated with Decreased Patient Survival

**Figure 3:**
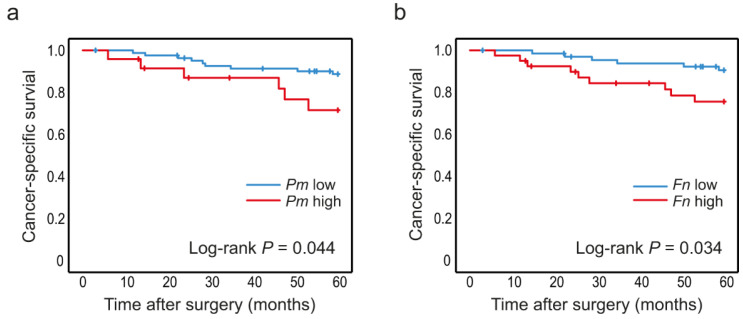
Levels of *P. micra* and *F. nucleatum* in tumour tissue in relation to CRC patient survival. Kaplan–Meier plots of 5-year cancer-specific survival for patients with high or low levels of (**a**) *P. micra* and (**b**) *F. nucleatum* in tumour tissue. Log-rank tests were used to calculate *p* values.

**Figure 4:**
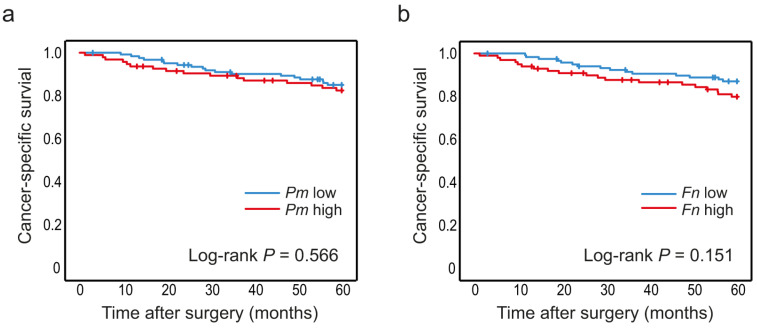
Levels of *P. micra* and *F. nucleatum* in faeces in relation to CRC patient survival. Kaplan–Meier plots of 5-year cancer-specific survival for patients with high or low levels of (**a**) *P. micra* and (**b**) *F. nucleatum* in faecal samples. Log-rank tests were used to calculate *p* values.

The levels of *P. micra* and *F. nucleatum* were analysed according to cancer-specific survival in CRC patients. Since no associations were found between the studied bacteria and stage, all stages (I–IV) of CRC patients were included to increase sample size. The median follow-up time from date of cancer surgery was 93.2 (1.2–134) months. Kaplan–Meier curves truncated at 5 years were estimated for patients with high and low levels of *P. micra* or *F. nucleatum* in tumour tissue (Figure 3) and in faeces (Figure 4). High levels of *P. micra* or *F. nucleatum* in tumour tissue were significantly associated with decreased patient survival (Figure 3). No significant differences in survival were found between patients with high and low levels of *P. micra* or *F. nucleatum* in faeces (Figure 4). When excluding stage IV CRC patiens from the analyses, *P. micra* continued to have a significant association with decreased survival (*p* = 0.031). For *F. nucleatum*, no significant association was found (*p* = 0.090).

High levels of *P. micra* in tumour tissue were also found to have a significant effect on survival in a multivariable analysis including tumour stage and grade (HR 3.82 (CI 1.12–13.0), *p* = 0.032), further strengthening the association between increased levels of *p. micra* in tumour tissue and decreased patient survival (Table 4). For *F. nucleatum*, the prognostic effect did not remain significant in the multivariable analysis (HR 2.70 (CI 0.83–8.80), *p* = 0.099) (Table 4).

## 4. Discussion

In this study, we investigated the presence of two CRC-associated microbes from the oral microflora, *P. micra* and *F. nucleatum*, in tumour tissue and in faeces of CRC patients in relation to prognosis. We found a novel association between high tumoural levels of *P. micra* and a decreased patient survival rate in CRC. High tumoural levels of *F. nucleatum* were also associated with decreased patient survival. We could further associate high levels of *P. micra* and/or *F. nucleatum* in tumour tissue with different patient clinicopathological and tumour molecular traits. In addition, we concluded that analyses of the tumour microbiota were more significant in terms of clinical and molecular associations than analyses of the faecal microflora.

When analysing levels of *P. micra* and *F. nucleatum* in tumour tissue in relation to tumour molecular features, high levels of both bacteria were found to be associated with *BRAF*-mutated tumours and tumours of the MSI subtype. Furthermore, *F. nucleatum* was significantly associated with right-sided tumours. The results are in line with earlier findings for *F. nucleatum* [19,29]. In a previous study by our group, as well as in a study by Purcell et al., associations between high levels of *P. micra* and *F. nucleatum* in tumour tissue and tumours of CMS subgroup 1 were found [22,25]. CMS 1 tumours are characterised by high immune infiltration and are commonly MSI, *BRAF*-mutated and right-sided, which is in line with the current findings [30]. High levels of *P. micra* and *F. nucleatum* in tumour tissue were further associated both with age under 59 and age above 80. In a study by Bhem et al., an association between high age and high levels of *F. nucleatum* in tumour tissue was found in gastric cancer patients [31]. However, *F. nucleatum* has also been associated with young onset CRC [32]. The non-linear association with age found in this study could partly depend on the association with tumours of the MSI subtype. These tumours are known to be more common in patients with hereditary Lynch syndrome and are likely to occur at a younger age [33]. In contrast, sporadic tumours of the MSI subtype are more common in older individuals, having had a longer time to develop mutations leading to MSI [33]. For *P. micra*, there was a significant association with the female sex, whereas for *F. nucleatum* there was a trend towards an association, although it was not significant. The association with the female sex has not previously been described and needs to be verified in other studies. However, right-sided tumours, tumours of the MSI subtype and *BRAF*-mutated tumours are known to be more frequent in older women [34].

Furthermore, we investigated associations between cancer-specific survival in colorectal cancer and levels of *P. micra* and *F. nucleatum* in tumour tissue. High levels of *P. micra* or *F. nucleatum* in tumour tissue were significantly associated with decreased five-year survival in stage I-IV CRC. This association remained significant in multivariable Cox regression analysis for *P. micra*. When excluding stage IV CRC patients, the association with decreased survival was still significant for *P. micra*. To our knowledge, this is the first study showing an association of *P. micra* in tumour tissue with decreased patient survival. However, one recent study has suggested a prognostic role of faecal *P. micra* in CRC [26]. *F. nucleatum*, on the other hand, has been studied more extensively and has previously been associated with decreased survival [14,15,16,17,18,19,20,21]. For example, in a large study by Mima et al. on tumour tissue samples from 1069 CRC cases within the Nurses’ Health Study and the Health Professionals Follow-Up Study, high levels of *F. nucleatum* were associated with decreased CRC-specific survival (HR 1.58, 95% CI 1.04–2.39) [19]. Interestingly, in this study, both *P. micra* and *F. nucleatum* were associated with tumours of the MSI subtype, generally known to have a better prognosis [35]. Associations were also found for *P. micra* and *F. nucleatum* with *BRAF* mutations, which have been proposed to partially mitigate the positive prognostic effect of MSI [35]. It is thus possible that tumours with *P. micra* and/or *F. nucleatum* colonisation represent a subgroup of MSI tumours with a worse prognosis. To strengthen this hypothesis, further subgroup analyses are needed in larger patient cohorts. In a study by Wei Z et al., high levels of *F. nucleatum* in tumour tissue were associated with a decreased survival rate and increased expression of TNF-α, suggesting that dysbiosis might worsen the patient’s prognosis by upregulating gut inflammation [14]. *F. nucleatum* has also been suggested to be associated with the development of tumour metastasis [36], and has in fact been detected in CRC liver metastases as well as the primary tumour [37]. Chen et al. have further proposed *F. nucleatum* to promote CRC metastasis through the regulation of caspase activation and recruitment domain 3 (CARD3, a prometastatic kinase), as well as activation of the nuclear factor-kappa B pathway resulting in migration of CRC cells [38,39]. In line with these findings, patients with tumours of the MSI subtype with metastatic spread are known to have decreased survival [35]. In addition, *F. nucleatum* has been associated with chemotherapy resistance through the regulation of autophagy pathways [40]. It is thus possible that the association with poor survival could depend on proinflammatory factors and the promotion of metastatic formation, as well as chemoresistance.

The levels of *P. micra* were found to be strongly associated with the levels of *F. nucleatum* in both tumour tissue and faeces, which is in line with two of our previous studies, as well as a study by Jun Yu et al. [12,23,25]. Interestingly, in a study by Horiuchi A et al., *P. micra* and *F. nucleatum* were shown to display synergistic effects in bacterial biofilm formation [41]. Drewes et al. also revealed an abundance of the human oral microbiota in right-sided tumours, including both *P. micra* and *F. nucleatum* [10], and right-sided tumours have been suggested to be associated with bacterial biofilm formation [42]. Thus, it is possible that high levels of the studied bacterial species are markers for a higher degree of dysbiosis and bacterial biofilm formation. In a study by Johnson et al., colonic mucosal biofilms were indeed suggested to affect cellular proliferation and tumour growth [43]. Further studies on the mechanistic role of *P. micra* and *F. nucleatum* in CRC prognosis are needed to support these theories.

In faecal samples, the associations of *P. micra* and *F. nucleatum* with clinical and molecular parameters were much weaker. *P. micra* and *F. nucleatum* were still associated with tumours of the MSI subtype. However, no association was found between *P. micra* or *F. nucleatum* and cancer-specific survival. One explanation for this could be that tumour tissue represents the local tumour microenvironment better than faecal samples do. In this study, we found significant associations between levels of *P. micra* and *F. nucleatum* in both tumour tissue and in faeces, respectively, suggesting that the faecal microflora still partly reflects the local tumour microflora. However, for more detailed analyses of the molecular role of *P. micra* and *F. nucleatum* in tumour progression and patient survival, tumour tissue appears to be the preferred choice.

One strength of this study is the inclusion of both tumour tissue and faecal samples originating from the same individuals, giving a better insight into the spatial presence of the examined species in association with studied traits. However, the lack of a validation cohort calls for further studies in order to confirm our results. Furthermore, our study does not include any mechanistic insights for the found associations. Thus, studies on the underlying mechanisms, including inflammatory and immunological processes, are needed in order to better understand the cause and effect relationships. Another area of interest is the association with dietary factors, which may directly and indirectly affect the constitution of the gut microbial content.

## 5. Conclusions

Our study suggests associations between high levels of *P. micra* and *F. nucleatum* in tumour tissue and decreased patient survival in CRC. We found that *P. micra* and *F. nucleatum* often colonise the same tumours, and that tumours colonised with *P. micra* and/or *F. nucleatum* more often are *BRAF*-mutated and of the MSI subtype. However, further studies on the role of the gut microbiota in CRC formation and progression, including underlying mechanisms, are needed. A better understanding of the role of gut microbial composition in CRC progression might contribute to future putative targets for therapy.

## Figures and Tables

**Table 1:** The distribution of patients with high and low levels of *P. micra* and *F. nucleatum* in tumour tissue and in faeces.

	Faeces					
	*P. micra*		*p* Value	*F. nucleatum*		*p* Value
**Fresh frozen tissue**	High	Low		High	Low	
***P. micra*, n (%)**						
High	20 (83.3)	4 (16.7)	<0.001	18 (75.0)	6 (25.0)	<0.001
Low	22 (26.2)	62 (73.8)		28 (34.1)	54 (65.9)	
***F. nucleatum*, n (%)**						
High	22 (55.0)	18 (45.0)	0.014	24 (61.5)	15 (38.5)	0.009
Low	20 (29.4)	48 (70.6)		23 (34.3)	44 (65.7)	

Fischer’s exact test was used to compare categorical variables.

**Table 2:** Clinicopathological characteristics of study patients in relation to *P. micra* and *F. nucleatum* in tumour tissue.

	*P. micra*		*p* Value	*F. nucleatum*		*p* Value
	Low	High		Low	High	
**Age, n (%)**						
≤59	11 (61.1)	7 (38.9)	0.023	7 (38.9)	11 (61.1)	0.017
60–69	32 (91.4)	3 (8.6)		28 (80.0)	7 (20.0)	
70–79	33 (80.5)	8 (19.5)		27 (65.9)	14 (34.1)	
≥80	10 (62.5)	6 (37.5)		8 (50.0)	8 (50.0)	
**Gender, n (%)**						
Male	53 (89.8)	6 (10.2)	0.002	43 (71.7)	17 (28.3)	0.073
Female	33 (64.7)	18 (35.3)		27 (54.0)	23 (46.0)	
**Location, n (%)**						
Right colon	23 (69.7)	10 (30.3)	0.269	12 (37.5)	20 (62.5)	0.002
Left colon	16 (76.2)	5 (23.8)		16 (76.2)	5 (23.8)	
Rectum	47 (83.9)	9 (16.1)		42 (73.7)	15 (26.3)	
**Stage, n (%)**						
I	29 (90.6)	3 (9.4)	0.171	23 (74.2)	8 (25.8)	0.425
II	28 (73.7)	10 (26.3)		24 (63.2)	14 (36.8)	
III	21 (70.0)	9 (30.0)		17 (54.8)	14 (45.2)	
IV	7 (77.8)	2 (22.2)		5 (55.6)	4 (44.4)	
**Tumour grade, n (%)**						
Low grade	71 (80.7)	17 (19.3)	0.143	58 (65.9)	30 (34.1)	0.207
High grade	13 (65.0)	7 (35.0)		10 (50.0)	10 (50.0)	
**Tumour type, n (%)**						
Non-mucinous	72 (79.1)	19 (20.9)	0.525	59 (64.8)	32(35.2)	0.416
Mucinous	12 (70.6)	5 (29.4)		9 (52.9)	8 (47.1)	

Fischer’s exact test was used to compare categorical variables.

**Table 3:** Molecular characteristics of study patients in relation to *P. micra* and *F. nucleatum* in tumour tissue.

	*P. micra*		*p* Value	*F. nucleatum*		*p* Value
	Low	High		Low	High	
***KRAS*** **status, n (%)**						
Wild type	51 (76.1)	16 (23.9)	0.638	43 (65.2)	23 (34.8)	0.691
Mutant	35 (81.4)	8 (18.6)		27 (61.4)	17 (38.6)	
***BRAF*** **status, n (%)**						
Wild type	75 (82.4)	16 (17.6)	0.030	62 (68.1)	29 (31.9)	0.039
Mutant	11 (57.9)	8 (42.1)		8 (42.1)	11 (57.9)	
**MSI status, n (%)**						
MSS	80 (83.3)	16 (16.7)	0.002	66 (68.8)	30 (31.3)	0.006
MSI	6 (42.9)	8 (57.1)		4 (28.6)	10 (71.4)	

Fischer’s exact test was used to compare categorical variables. Abbreviations: MSI, microsatellite. instable; MSS, microsatellite stable.

**Table 4:** 5-year survival uni- and multi-variable Cox analyses. The multivariable analyses were performed separately for *P. micra* and *F. nucleatum*.

	Univariable				Multivariable *P. micra*			Multivariable *F. nucleatum*		
	HR	95.0% CI		*p* Value	HR	95.0% CI	*p* Value	HR	95.0% CI	*p* Value
**Stage**										
I+II	1				1			1		
III	6.26	(2.04–19.2)		0.001	10.2	(1.23–84.62)	0.032	10.3	(1.23–86.2)	0.032
IV	34.07	(11.26–103.0)		<0.001	119	(12.8–1106)	<0.001	91.2	(10.0–828)	<0.001
**Gender**										
Female	1									
Male	1.21	(0.61–2.42)		0.584	NA		-	NA		-
**Age**										
≤59	1									
60–69	0.42	(0.18–1.02)		0.055	NA		-	NA		-
70–79	0.51	(0.21–1.22)		0.129	NA		-	NA		-
≥80	0.56	(0.18–1.80)		0.333	NA		-	NA		-
**Location**										
Right colon	1									
Left colon	0.81	(0.24–2.75)		0.731	NA		-	NA		-
Rectum	0.98	(0.42–2.27)		0.954	NA		-	NA		-
**Tumour grade**										
Low grade	1				1			1		
High grade	4.80	(2.24–10.28)		<0.001	3.16	(1.01–9.93)	0.048	2.73	(0.86–8.69)	0.089
**Tumour type**										
Non-mucinous	1									
Mucinous	2.10	(0.86–5.15)		0.103	NA		-	NA		-
***KRAS***										
Wild type	1									
Mutant	1.36	(0.58–3.17)		0.483	NA		-	NA		-
***BRAF***										
Wild type	1									
Mutant	1.35	(0.46–3.99)		0.589	NA		-	NA		-
**MSI**										
MSS	1									
MSI	0.78	(0.18–3.34)		0.739	NA		-	NA		-
**Tumour tissue**										
*P. micra* low	1				1					
*P. micra* high	2.77	(0.98–7.78)		0.054	3.82	(1.12–13.0)	0.032	NA		-
*F. nucleatum* low	1							1		
*F. nucleatum* high	2.91	(1.04–8.19)		0.043	NA		-	2.70	(0.83–8.80)	0.099
**Faeces**										
*P. micra* low	1									
*P. micra* high	1.22	(0.62–2.39)		0.567	NA		-	NA		-
*F. nucleatum* low	1									
*F. nucleatum* high	1.64	(0.83–3.22)		0.155	NA		-	NA		-

Variables included in the multivariable analyses were selected using 1. Backward Stepwise Conditional model and 2. only including variables with a significant effect on survival in the univariable analysis. Both methods resulted in the same variables being included in the multivariable analyses (stage, grade and levels of *P. micra* or *F. nucleatum* in tumour tissue). Abbreviation: NA, not applicable.

## Data Availability

The data presented in this study are available on reasonable request from the corresponding author.

## Supplementary Materials

The following supporting information can be downloaded at: https://www.mdpi.com/article/10.3390/cancers14235937/s1, Table S1: clinicopathological characteristics of study patients in relation to *P. micra* and *F. nucleatum* in faeces; Table S2: molecular characteristics of study patients in relation to *P. micra* and *F. nucleatum* in faeces.
