# Supplementary material for: Tumour Colonisation of Parvimonas micra Is Associated with Decreased Survival in Colorectal Cancer Patients
Source: Cancers (Basel). 2022 Nov 30;14(23):5937. doi: 10.3390/cancers14235937 (PMC9736682; doi:10.3390/cancers14235937)
Supplement: Supplementary file 1 [file cancers-14-05937-s001.zip › Supplementary Table S1.pdf]

Supplementary Table S1. Clinicopathological characteristics of study patients in relation to *P. micra* and *F. nucleatum* in faeces.

	P. micra		***P* value**	***F. nucleatum***		***P* value**
	Low	High		Low	High	
**Age, n (%)**						
≤ 59	27(64.3)	15(35.7)	0.510	26(61.9)	16(38.1)	0.584
60-69	48(52.2)	44(47.8)		45(50.0)	45(50.0)	
70-79	48(58.5)	34(41.5)		44(54.3)	37(45.7)	
≥ 80	15(50.0)	15(50.0)		15(48.4)	16(51.6)	
**Gender, n (%)**						
Male	86(58.1)	62(41.9)	0.512	72(49.7)	73(50.3)	0.192
Female	52(53.1)	46(46.9)		58(58.6)	41(41.4)	
**Location, n (%)**						
Right colon	30(60.0)	20(40.0)	0.739	27(54.0)	23(46.0)	0.916
Left colon	24(58.5)	17(41.5)		23(56.1)	18(43.9)	
Rectum	84(54.2)	71(45.8)		80(52.3)	73(47.7)	
**Stage, n (%)**						
I	30(63.8)	17(36.2)	0.562	31(66.0)	16(34.0)	0.144
II	44(53.7)	38(46.3)		45(54.9)	37(45.1)	
III	36(52.2)	33(47.8)		33(49.3)	34(50.7)	
IV	23(60.5)	15(39.5)		16(42.1)	22(57.9)	
**Tumour grade, n (%)**						
Low grade	108(58.4)	77(41.6)	0.684	105(57.4)	78(42.6)	0.311
High grade	15(53.6)	13(46.4)		13(46.4)	15(53.6)	
**Tumour type, n (%)**						
Non-mucinous	110(57.9)	80(42.1)	0.394	106(56.1)	83(43.9)	0.388
Mucinous	12(48.0)	13(52.0)		11(45.8)	13(54.2)	

Fischer´s exact test was used to compare categorical variables.
