# Supplementary material for: Tumour Colonisation of Parvimonas micra Is Associated with Decreased Survival in Colorectal Cancer Patients
Source: Cancers (Basel). 2022 Nov 30;14(23):5937. doi: 10.3390/cancers14235937 (PMC9736682; doi:10.3390/cancers14235937)
Supplement: Supplementary file 1 [file cancers-14-05937-s001.zip › Supplementary Table S2.pdf]

Supplementary Table S2. Molecular characteristics of study patients in relation to *P. micra* and *F. nucleatum* in faeces.

	P. micra		***P* value**	***F. nucleatum***		***P* value**
	Low	High		Low	High	
***KRAS* status, n (%)**						
Wild type	69(57.5)	51(42.5)	0.330	63(53.4)	55(46.6)	0.522
Mutant	39(66.1)	20(33.9)		35(59.3)	24(40.7)	
***BRAF* status, n (%)**						
Wild type	95(62.1)	58(37.9)	0.215	87(57.6)	64(42.4)	0.153
Mutant	14(48.3)	15(51.7)		12(41.4)	17(58.6)	
**MSI status, n (%)**						
MSS	104(64.2)	58(35.8)	0.004	93(58.1)	67(41.9)	0.037
MSI	6(28.6)	15(71.4)		7(33.3)	14(66.7)	

Fischer´s exact test was used to compare categorical variables. *Abbreviations*: MSI, microsatellite unstable; MSS, microsatellite stable.
